# Expansion and diversification of the MSDIN family of cyclic peptide genes in the poisonous agarics *Amanita phalloides* and *A. bisporigera*

**DOI:** 10.1186/s12864-016-3378-7

**Published:** 2016-12-15

**Authors:** Jane A. Pulman, Kevin L. Childs, R. Michael Sgambelluri, Jonathan D. Walton

**Affiliations:** 1 Department of Plant Biology, Michigan State University, East Lansing, MI 48824 USA; 2 Center for Genomics-Enabled Plant Science, Michigan State University, East Lansing, MI 48824 USA; 3 Department of Biochemistry and Molecular Biology, Michigan State University, East Lansing, MI 48824 USA; 4 Department of Energy Plant Research Laboratory, Michigan State University, East Lansing, MI 48824 USA

**Keywords:** Amatoxin, Amanitin, Phallotoxin, Phalloidin, Phallacidin, Poisonous mushroom, Cyclic peptide, Cycloamanide, Antamanide

## Abstract

**Background:**

The cyclic peptide toxins of *Amanita* mushrooms, such as α-amanitin and phalloidin, are encoded by the “MSDIN” gene family and ribosomally biosynthesized. Based on partial genome sequence and PCR analysis, some members of the MSDIN family were previously identified in *Amanita bisporigera*, and several other members are known from other species of *Amanita*. However, the complete complement in any one species, and hence the genetic capacity for these fungi to make cyclic peptides, remains unknown.

**Results:**

Draft genome sequences of two cyclic peptide-producing mushrooms, the “Death Cap” *A. phalloides* and the “Destroying Angel” *A. bisporigera*, were obtained. Each species has ~30 MSDIN genes, most of which are predicted to encode unknown cyclic peptides. Some MSDIN genes were duplicated in one or the other species, but only three were common to both species. A gene encoding cycloamanide B, a previously described nontoxic cyclic heptapeptide, was also present in *A. phalloides*, but genes for antamanide and cycloamanides A, C, and D were not. In *A. bisporigera*, RNA expression was observed for 20 of the MSDIN family members. Based on their predicted sequences, novel cyclic peptides were searched for by LC/MS/MS in extracts of *A. phalloides*. The presence of two cyclic peptides, named cycloamanides E and F with structures cyclo(SFFFPVP) and cyclo(IVGILGLP), was thereby demonstrated. Of the MSDIN genes reported earlier from another specimen of *A. bisporigera*, 9 of 14 were not found in the current genome assembly. Differences between previous and current results for the complement of MSDIN genes and cyclic peptides in the two fungi probably represents natural variation among geographically dispersed isolates of *A. phalloides* and among the members of the poorly defined *A. bisporigera* species complex. Both *A. phalloides* and *A. bisporigera* contain two prolyl oligopeptidase genes, one of which (POPB) is probably dedicated to cyclic peptide biosynthesis as it is in *Galerina marginata*.

**Conclusion:**

The MSDIN gene family has expanded and diverged rapidly in *Amanita* section *Phalloideae*. Together, *A. bisporigera* and *A. phalloides* are predicted to have the capacity to make more than 50 cyclic hexa-, hepta-, octa-, nona- and decapeptides.

**Electronic supplementary material:**

The online version of this article (doi:10.1186/s12864-016-3378-7) contains supplementary material, which is available to authorized users.

## Background

The characteristic toxins of poisonous agarics (mushrooms; Agaricales) in the genus *Amanita* include the amatoxins such as α-amanitin and the phallotoxins such as phalloidin. Both families of toxins are bicyclic peptides biosynthesized on ribosomes as precursor peptides [1]. These were the first ribosomally encoded post-translationally modified peptides (RiPPs) to be described from the Kingdom Mycota [2]. Additional fungal RiPPs have subsequently been discovered in filamentous fungi (Ascomycota) [3–5].

From a partial genomic sequence obtained by 454 pyrosequencing, it was shown that the genes for α-amanitin and phallacidin belong to a family of at least 15 genes in *Amanita bisporigera* (*Ab*) called the “MSDIN” family for the first five conserved amino acids in the precursor peptides [1]. The MSDIN precursor peptides are 33–37 amino acids in length and comprise two conserved regions, a 10-amino acid “leader” and a 17-amino acid “follower”, flanking a highly variable “core” region of 6–10 amino acids that contains the amino acids present in the mature toxins.

Subsequent to its discovery in *Ab*, the MSDIN family has been found in other cyclic peptide toxin-producing species of *Amanita*, including *A. ocreata, A. phalloides* (*Ap*), and *A. exitialis* [1, 6]. The MSDIN family is absent from fungi that do not produce amatoxins or phallotoxins, including species of *Amanita* outside section *Phalloideae* [1]. *Galerina marginata* (*Gm*), an agaric not closely related to *Amanita*, also produces α-amanitin on ribosomes but does not possess an extended gene family [7, 8]. The α-amanitin gene in *Gm* is the same length (35 amino acids) as the homolog in *Ab*, but its primary amino acid sequence is divergent outside the core region [7].

The initial post-translational processing step of the α-amanitin precursor peptide in *Gm* is catalyzed by GmPOPB, a specialized member of the prolyl oligopeptidase family of serine proteases [9]. POPB first cleaves at the carboxy side of a highly conserved Pro residue at the C-terminus of the 10-amino acid leader sequence and then transpeptidates at a second Pro (which remains in the final product) to produce a homodetic cyclic octapeptide. Studies with synthetic peptides have elucidated the general sequence and structural requirements of the precursor peptide for processing by POPB [9]. A dedicated POP most likely also processes the cyclic peptide precursor peptides in *Amanita* [10].

In order to more fully understand the genomic potential for cyclic peptide production by *Amanita* section *Phalloideae*, we have generated draft genome sequences of *Ap* and *Ab*. We show that each species contains ~30 members of the MSDIN family, only three of which are in common between the two fungi. Furthermore, two specimens within the *A. bisporigera* species complex have different complements of MSDIN genes. These results illuminate the deep genetic potential of the *Amanita* toxin biosynthetic pathway to produce a range of modified and unmodified cyclic peptides by the same biosynthetic pathway.

## Results

### Genome and transcriptome assembly of *Ap* and *Ab*

The sequencing and assembly statistics for the genomes for *Ap* and *Ab* are shown in Table 1. After an initial assembly of the *Ap* genome, Blobology analysis identified 18 contigs that were contaminated with non-fungal reads [11]. The raw reads associated with the contaminated contigs were removed before reassembling the genome. The genome assembly of *Ap* contained 1465 contigs greater than 1 kb and a total size of ~40 Mb with an N50 scaffold size of 54 kb. Using a basidiomycete single-copy core protein database, analysis with BUSCO [12] resulted in the identification of 92% complete and 3.6% fragmented protein sequences in the *Ap* genome assembly. Only 4.3% of the core basidiomycete core proteins were missing.

**Table 1:** Basic statistics for the genome assemblies

	*A. phalloides*	*A. bisporigera*
Assembly size	40 Mb	75 Mb
Predicted fold coverage	69X	74X
Number of contigs	5,437	23,572
Contig N50	21,781 bp	5,866 bp
Number of Scaffolds	1,465	10,390
Scaffold N50	54,288 bp	13,906 bp
Coverage of complete BUSCO basidomycete genes	92%	73%

For *Ab*, analysis of an initial genome assembly with Blobology indicated that 163 contigs were contaminated with non-fungal sequences. Raw reads aligned to these sites of contamination were removed, and the remaining reads were reassembled. The final *Ab* genome assembly had a total size of ~75 Mb with 10,390 scaffolds with an N50 scaffold size of 13.9 kb (Table 1). The genome contained 73% of the complete proteins in the BUSCO basidiomycete single-copy core protein dataset, 9.2% of fragmented proteins, and was missing 17% of the core proteins. Transcript assembly of *Ab* RNA-seq reads with Trinity yielded 67,879 transcripts, including isoforms.

### MAKER annotation of *Ap* and *Ab* genomes

The annotation of *Ap* resulted in 10,221 gene models of which 8177 were supported by protein alignments and/or known Pfam domains (Additional file 1: Table S1) [13, 14]. These gene models were used as the high-quality gene set for functional annotation and downstream ortholog analysis. The average predicted transcript length was 1494 bp, with a unimodal GC distribution ranging from 31 to 68 with a peak at 49 (Additional file 1: Figure S1).

The annotation of *Ab* resulted in 22,189 gene models of which 14,886 were supported by transcript alignments, protein alignments, and/or known Pfam domains (Additional file 1: Table S1). This set of 14,886 gene models was retained as the high-quality gene set for functional annotation and downsteam ortholog analysis. The average transcript length was 1182 bp, with a unimodal GC distribution ranging from 17–67 with a peak at 48 (Additional file 1: Figure S1).

### MSDIN gene search and annotation

No MSDIN genes were annotated by the MAKER annotation pipeline even after the minimum protein length parameter was reduced to 150 bp. We also trained SNAP and AUGUSTUS to find short genes by using only short genes in the training sets, but this also resulted in no MSDIN gene predictions (data not shown). Finally, by using known members of the MSDIN family as tblastn queries, 33 MSDIN genes were manually identified in *Ap*, of which 29 were unique (Table 2). The predicted proteins of 23 of them started with the canonical sequence “MSDIN”, and the others with some single amino-acid variant of MSDIN, i.e., MSDMN, MSDVN, MSDIK, MSEIN, MSDTN or MSNIN. All had the two canonical Pro residues required for processing by prolyl oligopeptidase B (POPB), except one predicted protein (Apha_msdin_31) was missing the Pro residue immediately upstream of the variable region and another (Apha_msdin_30) was missing the second Pro residue [9]. The latter precursor peptide sequence also lacked the terminal Cys residue that is probably required for processing by POPB [9]. Among the *Ap* MSDIN genes were three (Apha_msdin_26, 29, and 33) encoding phalloidin (AWLATCP) from three separate scaffolds. Two genes (Apha_msdin_12 and 14) encoded β-amanitin (IWGIGCDP). There were single genes for phallacidin and α-amanitin (Apha_msdin_1 and 13, respectively). The Apha_msdin_27 gene encoded cycloamanide B, which is an unmodified monocyclic heptapeptide of sequence SFFFPIP [15].

**Table 2:**
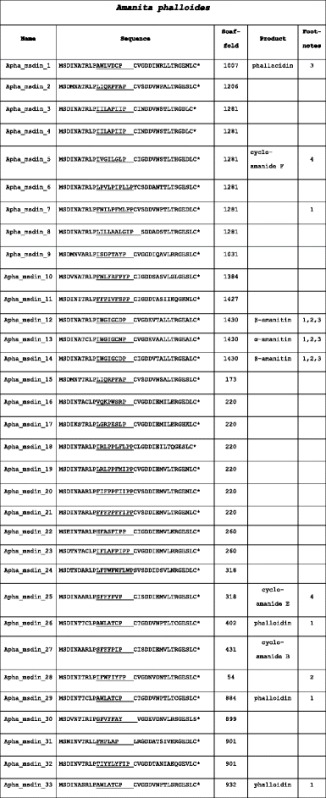
Predicted peptide sequences of the MSDIN family members in *A. phalloides* (*Ap*)

Core regions are underlined. Spaces were introduced after some of the core sequences to emphasize alignments of the follower sequences

Notes:

1. Core region previously reported from *A. bisporigera* [1]

2. Core region previously reported from *A. exitialis* [6]

3. Core region previously reported from *A. rimosa* [18]

4. First reported in this paper

*indicates a stop codon

The MSDIN genes from *Ap* were found in many clusters on numerous scaffolds. Scaffold_220 contained six MSDIN genes, all of which had different core sequences (Apha_msdin_16 through Apha_msdin_21) (Table 2). Three scaffolds (Scaffold_260, Scaffold_318, and Scaffold_901) each had two unique MSDIN genes. Scaffold_1281 had six MSDIN genes; two (Apha_msdin_3 and 4) had identical core regions (IILAPIIP), and four others, Apha_msdin_5 through Apha_msdin_8, were unique. The three genes encoding α-amanitin and β-amanitin were clustered on Scaffold_1430.

The MSDIN genes from *Ab* also required manual annotation. The *Ab* genome assembly contained 31 MSDIN genes of which 27 were unique (Table 3). The predicted proteins of 24 *Ab* MSDIN genes started with the canonical “MSDIN” sequence. The first Pro residue adjacent to the variable region of these predicted MSDIN genes was conserved in all of the predicted proteins, but three (Abis_msdin_17, 18, and 27) lacked the second Pro residue. Additionally, four gene products (Abis_msdin_7, 11, 30, and 31) did not contain a terminal Cys residue that is essential for cyclization by POPB (Luo et al. [9]) but instead contained the similar amino acid Ser.

**Table 3:**
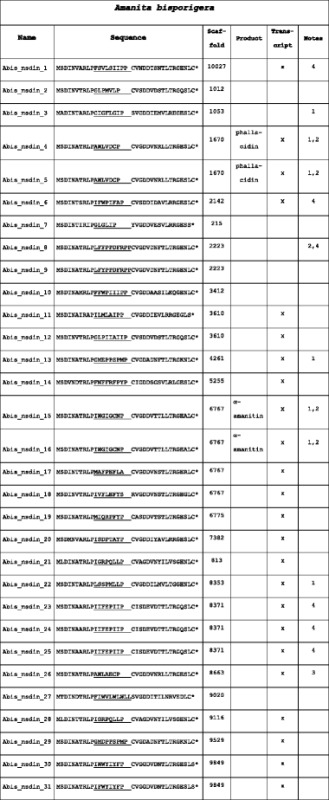
Predicted peptide sequences of the MSDIN family members in *A. bisporigera* (*Ab*)

Core regions are underlined. Spaces were introduced after some of the core sequences to emphasize alignments of the follower sequences. “Transcript” indicates whether the sequence was found by RNAseq in the transcriptome of *Ab*

Notes:

1. Core region previously reported from *A. bisporigera* [1]

2. Core region previously reported from *A. exitialis* [6]

3. Core region previously reported from *A. rimosa* [18]

4. Core region previously reported from *A. bisporigera* [26]

*indicates a stop codon

Many of the *Ab* MSDIN genes were clustered in the genome (Table 3). Abis_msdin_4 and 5, both encoding phallacidin, were found on Scaffold_1670. Scaffold_6767 had four MSDIN genes including Abis_msdin_15 and 16, both of which encode α-amanitin. Three genes with identical core sequences (IIFEPIIP) were found on Scaffold_8371. Two genes on Scaffold_9849 (Ab_msdin_30 and 31), encoded nearly identical core regions (IWWYIYFP and IFWYIYFP). Scaffold_3610 contained two MSDIN genes with dissimilar core sequences, Abis_msdin_11 and 12.

In both *Ap* and *Ab*, a number of additional sequences were identified that showed some similarity to the MSDIN family but that were truncated or considered excessively divergent to be conclusively identified as members of the family. However, of possible significance to the evolution of the MSDIN gene family, two nearly identical sequences in *Ab* lacked any core sequences whatsoever (MSGINAARLP/AVGDDVEMVLRRGKR and MSGINAARLP/AVGDDVEMVLRRGER; the slash indicates where the core sequence would be). Despite the similarities to MSDIN genes, these loci were left unannotated.

### Orthology between *Ap, Ab*, and *A. muscaria*

*Ap* and *Ab* are both in sect. *Phalloideae* of subgenus *Lepidella*, and *A. muscaria* is in sect. *Amanita* of subgenus *Amanita* [16]. The orthology analysis resulted in a total of 7843 ortholog groups of which 4464 contained at least one protein from each of the three species. There were 892 ortholog groups that contained proteins from only *Am* and *Ap*, 591 groups with proteins only from *Am* and *Ab*, and 379 groups consisting of proteins only from *Ap* and *Ab.* The remaining 1517 groups each contained proteins from only one species. Both the POP genes and the MSDIN genes were clustered within the ortholog groups (Additional file 1: Table S2). One group containing eight proteins included both POPA and POPB from both *Ap* and *Ab* and one protein from *A. muscaria* (jgi|Amamu1|74086|e_gw1.11.99.1) (Additional file 1: Table S2). This protein is annotated as a prolyl oligopeptidase by JGI and a blastp search against the NCBI nr database shows it shares 77% identity with POPA from *Ab* (ADN19204.1) and 66% identity with POPA from *Gm* (AEX26937.2). The *A. muscaria* gene is therefore probably the ortholog of POPA, the “housekeeping” POP (see below).

An additional twelve orthologous groups were found to contain MSDIN proteins. Seven of these contained proteins from a single species (Additional file 1: Table S2). One of these groups contained two copies of phalloidin in *Ap* (Apha_msdin_26 and 29). In another of these single species ortholog groups, cycloamanide B (Apha_msdin_27) was predicted to be an ortholog of Apha_msdin_25, the only difference being a single amino acid subsitution (SFFFPVP to SFFFPIP) in the core region. The remaining five ortholog groups all contained proteins from both *Ab* and *Ap*. One contained two identical core regions, Apha_msdin_9 and Abis_msdin_20, a presumptive peptide shared between the species. One group contained three identical core regions identified as phallacidin, two from *Ab* and one from *Ap*. α-Amanitin in *Ap* (Apha_msdin_15) grouped with one MSDIN from *Ab* (Abis_msdin_19) as well as a second *Ap* protein (Apha_msdin_2). The final two groups contained orthologs from both *Ab* and *Ap* with one or more differences within the core regions.

### Comparison of the MSDIN family in *Ap*, *Ab*, and other *Amanita* species

*Ap* and *Ab* together contained a total of 64 MSDIN sequences representing 54 unique core regions (Tables 2 and 3). Only α-amanitin, phallacidin, and one unnamed predicted octapeptide (Aph_msdin_9 and Abis_msdin_20, with core region sequence ISDPTAYP) were common between *Ab* and *Ap*. Overall, *Ap* has 29 non-duplicate core regions and *Ab* has 27.

Tryptathione, the cross-bridge formed between Trp and Cys, is a hallmark of the amatoxins and phallotoxins [17]. Only one gene (Abis_msdin_26) encodes a novel peptide capable of containing tryptathionine, i.e., core sequence AWLAECP. It differs by one amino acid from phalloidin, having Glu instead of Thr at amino acid #5 [15]. In having an acidic residue at position #5, it resembles phallacidin. This core sequence was also present in *Amanita rimosa* [6]. All of the other MSDIN genes probably encode monocyclic peptides.

The MSDIN family in *Ap* and *Ab* has some overlap with the MSDIN family in *A. exitialis*, a mushroom implicated in multiple human poisonings in China [18]. In addition to α-amanitin, β-amanitin, and phallacidin, the transcriptome of *A. exitialis* contains genes encoding LFFPPDFRPP (Abis_msdin_8) and IFWFIYFP (Apha_msdin_28). Neither *Ab* nor *Ap* contains a gene for the nonapeptide amanexitide, cyclo(VFSLPVFFP), which was isolated from *A. exitialis* [18, 19].

### Expression of the novel MSDIN family members

To determine whether any of the novel MSDIN family members were expressed, RNASeq was performed with mRNA extracted from *Ab* basidiocarps. Transcripts for 20 unique (i.e., counting duplicated genes only once) MSDIN sequences were detected (Table 3), including phallacidin and α-amanitin. In every case, the transcript sequences confirmed the presence of an intron interrupting the fourth from the last codon (including the stop codon). The strong consensus for the last two amino acids of the MSDIN family precursor peptides was Leu-Cys (see below).

It was of particular interest whether any of the novel MSDIN genes were expressed at the level of actual cyclic peptides. *Ap* is known to produce cyclic peptides other than the bicyclic amatoxins and phallotoxins [15]. These include the cycloamanides and antamanide [20]. The four known cycloamanides are monocyclic hexa-, hepta-, or octapeptides, and antamanide is a monocyclic decapeptide. Other than being cyclized, none have the post-translational modifications characteristic of the amatoxins and phallotoxins such as tryptathionine bridge formation, hydroxylation, and α-carbon epimerization. Although the cycloamanides are considered to be non-toxic to mammals, some have immunosuppressive activity, and antamanide (cyclo[FFVPPAFFPP]) protects mice against the toxic effects of phallotoxins and blocks mitochondrial pore formation [15, 21, 22]. *Ap* had a gene (Apha_msdin_27) encoding cycloamanide B, cyclo(SFFFPIP). We did not find genes for any of the other cycloamanides or antamanide in *Ap* or *Ab*. However, predicted decapeptides related to antamanide were present in both *Ap* and *Ab. Ap* contained FFFPPFFIPP (Apha_msdin_21), IRLPPLFLPP (Apha_msdin_18), LRLPPFMIPP (Apha_msdin 19), and FIFPPFIIPP (Apha_msdin_20). *Ab* contained FFQPPEFRPP [1] and LFFPPDFRPP (Abis_msdin_8) and LFYPPDFRPP (Abis_msdin_9). The consensus for these seven decapeptide sequences (XXXPPXXXPP) contains two pairs of Pro residues.

To determine if any of the novel predicted peptides were produced, extracts of *Ap* were fractionated by high performance LC, and the masses of the unknown cyclic peptides in Table 2 were monitored by mass spectrometry (MS). Masses corresponding to the cyclized versions with zero to four hydroxylations were extracted based on analogy to the phallotoxins and amatoxins, which can each have up to four hydroxylations.

α-Amanitin, β-amanitin, phalloidin, phallacidin, and several minor toxins (γ-amanitin, amanin, phallisacin, and others) were detectable by LC/MS in *Ap* extracted by the standard 50% methanol procedure [23]. All of the known amatoxins and phallotoxins can be encoded by just four genes, corresponding to core peptide sequences IWGIGCNP, IWGIGCDP, AWLVDCP, and AWLATCP [1, 23]. No compounds with the predicted masses of any of the hypothetical peptides with novel core sequences were detected in the methanol extracts except one compound of M + H^+^ of m/z 893, which could correspond to cyclo(ISDPTAYP) with three hydroxylations. On the basis of its mass, this compound was purified by two steps of reverse phase chromatography. Its relative UV absorbances at 257, 280, 295, and 305 nm were consistent with the presence of Tyr. However, the high resolution monoisotopic mass of the compound as determined by ToF/MS was 892.3201, giving a most probable elemental formula of C_34_H_52_N_8_O_20_. The elemental composition of the putative trihydroxylated derivative of cyclo(ISDPTAYP) would be C_39_H_56_N_8_O_16_, giving a monoisotopic mass of 892.3814. The mass discrepancy (68 ppm), supported by the relative heights of the ^13^C/^12^C signals, precluded the novel compound from being the predicted cyclic peptide. Elucidation of the structure of the compound of m/z 892.32 is in progress.

Many of the predicted novel peptides have a preponderance of hydrophobic amino acids, and the cycloamanides were originally found in the lipophilic extracts of *Ap* [15, 20]. Hydrophobicity is an important pharmacokinetic property because it affects both the water solubility and the membrane permeability of a peptide. Therefore, ethanol/chloroform extracts of *Ap* were also analyzed by LC/MS. Nominal masses corresponding to some of the predicted unmodified cyclized peptides in Table 2 were detected. One of m/z 822.4 could correspond to Apha_msdin_25, cyclo(SFFFPVP), which differs by one amino acid from cycloamanide B. This compound was analyzed in more detail by MS/MS and mMass [24]. The elemental composition (C_45_H_56_N_7_O_9_) was consistent with this structure (predicted m/z 822.4190; m/z observed 822.4218; 3.4 ppm mass discrepancy). Furthermore, the MS/MS fragmentation pattern and analysis by mMass strongly supported the predicted amino acid composition and sequence, i.e., sequential loss of Val, Pro, Phe, Phe, and Phe and giving overlapping peptide fragments for full coverage of the cyclic backbone (Additional file 1: Figure S2). This new compound, predicted from the genome sequence, has been named cycloamanide E. LC/MS/MS also indicated the presence of another novel cycloamanide, Apha_msdin_5, with a structure of cyclo(IVGILGLP). This compound, eluting at 14.97 min, had a mass of 763.5118 (predicted m/z 763.5076, 5.5 ppm mass discrepancy) and mMass analysis indicated the expected amino acid composition and sequence (Additional file 1: Figure S3).

### Amino acid distribution in the core regions

**Fig. 1:**
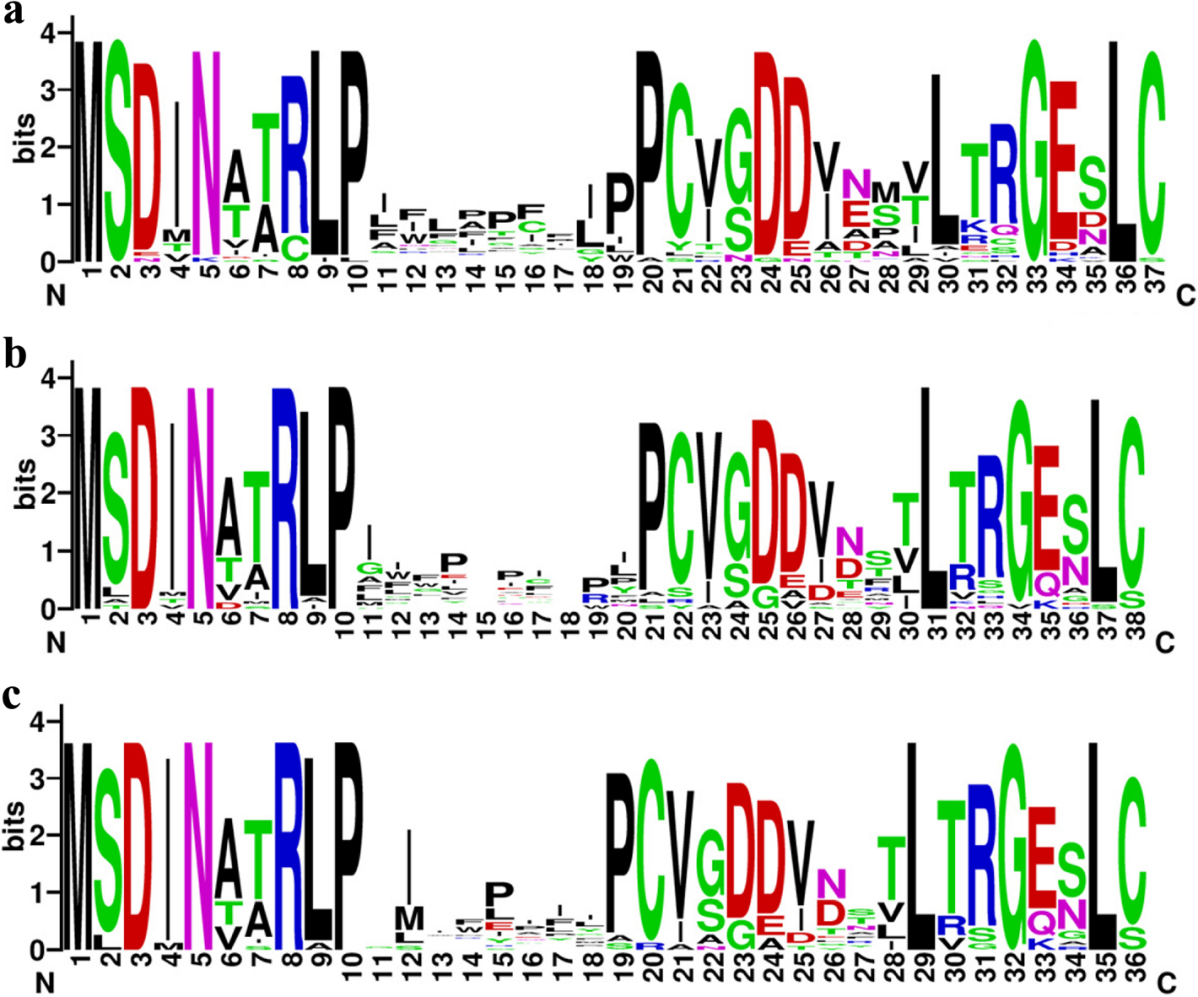
WebLogo [48] alignment of MSDIN sequences from the genomes of **a** *A. phalloides* and **b** *A. bisporigera*. **c** Alignment of predicted precursor peptides from the transcriptome of *A. bisporigera*

As previously reported for *Ab* based on its partial genome sequence [1, 25], the core regions of the MSDIN family in the full genomic complement of both *Ab* and *Ap* were more variable than the leader and follower sequences (Fig. 1). The predicted core peptides ranged in size from 6 to 10 amino acids with a mean of 8.2 in *Ap* and 8.4 in *Ab* and a mode of 8 in both (Tables 2 and 3).

**Fig. 2:**
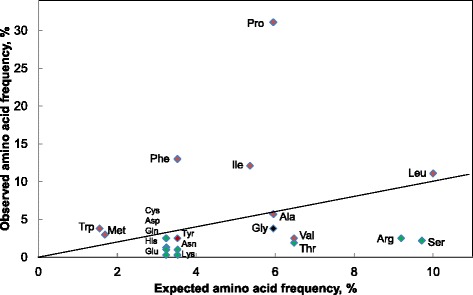
Observed vs. expected amino acid distribution in the core regions of the MSDIN peptides from *Ap*. Hydrophobic amino acids are shown in red and polar/charged amino acids in green. The line indicates a slope = 1 (no bias). Additional file 1: Table S3 shows the results for *Ab*

The distribution of amino acids in the core regions of the MSDIN family were similar in *Ab* and *Ap* (Additional file 1: Table S3). Every proteinogenic amino acid was found in at least one MSDIN family member. Pro was the most abundant amino acid in the core regions of both species. This was due to not just the conserved terminal Pro required for processing by POPB but also to a disproportionately high number of internal Pro residues. There was also an overall bias towards the hydrophobic amino acids Ile and Phe and against charged and polar residues such as Thr, Arg, and Ser (Fig. 2).

### Conservation in the leader and follower sequences

In addition to the two Pro residues required for processing by POPB, two Leu residues in the C-terminus, seven residues apart, are highly conserved in the MSDIN precursor peptides (Fig. 1). Although the C-terminus of the α-amanitin gene (*AMA1*) of *Gm* is highly divergent from that of *Ab*, both have Leu and either Leu (in the case of *Ab*) or the similar amino acid Ile (in the case of *Gm*) at the same positions [7]. These observations are consistent with the importance of an amphipathic α-helix in the C-terminal portion of the precursor peptide for correct cyclization by POPB, as was hypothesized [9].

Changing the terminal Cys to Ala inhibits cyclization by GmPOPB of the α-amanitin precursor peptide GmAMA1 [9]. Several of the precursor peptides predicted end in Ser rather than the canonical Cys (Tables 2 and 3, Fig. 1). However due to their chemically similar side chains it is possible that Ser can functionally subsitute for Cys.

**Table 4:**
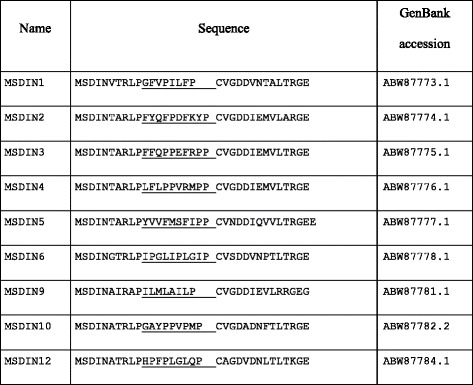
MSDIN peptides previously reported from the genome of *Ab* [1] but not found in the current genome sequence. ILMLAILP (#6) was also reported by Zhou et al. [26] from *Ab*

### Determination of the internal transcribed spacer (ITS) region sequences in *Ab* and *Ap*

The ITS sequences of both *Ab* and *Ap* were obtained from the genome sequences using the ITS sequence of *Ab* as query (Additional file 1: Figures S4 and S5). The *Ap* ITS sequence showed 100% identity with GenBank accessions GQ250407, EU909444, KF535946, GQ221841, and U909441, as well as many others, all of which are annotated as *A. phalloides*. The ITS region of the specimen of *Ab* sequenced in the current work was 100% identical to GenBank accessions GQ166893 and KJ638292, both of which are annotated as *A. bisporigera*, and 99% identical to KP221303, KJ466421, and several others annotated as *A. suballiacea*. The ITS region was also 98–99% identical to GenBank accessions annotated as *Amanita* sp. ‘sp-O01’, *Amanita* sp. ‘5 ZLY-2014’, *A. virosa*, or *A. ocreata*. Most of these sequences and the specimens from which they were obtained have not been described outside of GenBank. The ITS of the specimen of *Ab* sequenced in this paper was 92% identical to the ITS sequence of the specimen of *Ab* sequenced earlier [1], which was deposited in GenBank in 2004 with accession number AY550243. Since then, many additional *Amanita* ITS sequences have been deposited in GenBank. As of this writing, AY550243 shows 99–100% identity with sequences annotated as *A. bisporigera* (KR919771 and KR919772), *A. virosa* (HQ539860 and HQ539756), *A. phalloides* (HQ539826, HQ539722, and DQ071721), *A. marmorata* (HQ539813), and *A. verna* (HQ539859). That is, there appear to be many discrepancies between ITS sequences and fungal nomenclature in regard to *Ab* and its close relatives.

### MSDIN gene family differences within *Ab*

Based on a partial genome sequence, 14 members of the MSDIN family were earlier described from *Ab* [1, 26]. The genome of *Ab* described in the current work has 31 total and 27 unique MSDIN family members (Table 3). Surprisingly, 9 of the 14 sequences described in Hallen et al. [1] were not found in the current genome sequence (Table 4). In contrast, all eight of the previously described MSDIN sequences from *Ap* were present in the current *Ap* genome [1, 6]. A possible explanation for the result with *Ab* comes from consideration of the relatively low identity of the ITS regions (92%) of the specimen sequenced here compared to the one sequenced earlier [1]. That is, despite their morphological similarity and having been collected in the same location, the specimen sequenced earlier and the one sequenced in this paper are sufficiently different that it is reasonable to consider them to be distinct species, and this is reflected in their different complement of MSDIN sequences.

### Annotation and analysis of prolyl oligopeptidase (POP) genes

The initial post-translational processing of the MSDIN precursor peptides is catalyzed in *Gm* by a specialized prolyl oligopeptidase called POPB [9, 25]. GmPOPB differs from GmPOPA and from other POPs from other organisms in several respects. First, GmPOPB can act on larger peptides (at least 35 amino acids) compared to other POPs, which are limited to peptides of ~30 amino acids. Second, it poorly utilizes the model substrate *Z*-Gly-Pro-*p*-nitroanilide compared to other POPs. Third, it can catalyze not only peptide bond hydrolysis but also transpeptidation, thereby converting the 35-amino acid α-amanitin precursor peptide to cyclo(IWGIGCNP) [9].

The intron/exon structures of the POPA and POPB genes of *Ab* (*AbPOPA* and *AbPOPB*, respectively) had been experimentally determined previously [10]. Predicted POPA and POPB proteins from *A. muscaria* and *Ab* were aligned to the draft *Ab* and *Ap* genome assemblies, and these alignments along with SNAP and AUGUSTUS *ab initio* gene predictions were used to guide manual annotation of the *Ap* POP genes. *ApPOPA* was found on Scaffold_578, and *ApPOPB* on Scaffold_897. *AbPOPA* was found on Scaffold_8964. *AbPOPB* was on Scaffold_1287, but the underlying sequence contains several gaps. The POP gene models were added to the final annotation, protein, and transcript files.

**Fig. 3:**
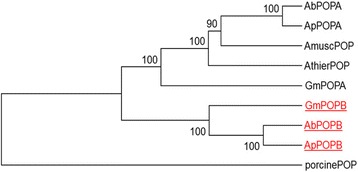
Phylogenetic tree based on alignment of POP proteins from species of *Amanita*, *Galerina marginata*, and porcine (*Sus scrofa*). Ap, *A. phalloides*; Ab*, A. bisporigera*; Amusc, *A. muscaria*; Athier*, A. thiersii*; Gm, *G. marginata*. GenBank acession numbers of AbPOPA, AbPOPB, GmPOPA, and GmPOPB are ADN19204, ADN19205, AEX26937, and AEX26938, respectively. JGI identifiers for AmuscPOP and AthierPOP are Amamu1|74086 and Amath1|193040, respectively. POPB proteins are in red and underlined. Protein alignments and tree construction were performed using UPGMA within ClustalW and 500 bootstrap replicates in MEGA version 6 [45]

Searching all Ascomycota and Basidiomycota sequences in the JGI databases using BLASTP and either POPA or POPB as queries indicated that POP genes are present throughout the Basidiomycota but there are no clear POP homologs in any species in the Ascomycota. The lack of POP genes in the Ascomycota is surprising because POPs are clearly present in all other branches of life, including bacteria, plants, and animals [27]. Most Basidiomycota have a single POP gene, including *A. muscaria* and *A. thiersii*, which do not have the MSDIN gene family and do not produce known cyclic peptide toxins. However, the cyclic peptide-producing fungi that have been sequenced (i.e., *Ab*, *Ap*, and *Gm*) each have two POPs [9, 10]. When aligned, the POPAs of the toxin-producing fungi and the single POP proteins of *A. muscaria* and *A. thiersii* formed one clade and the POPBs formed another (Fig. 3). This is consistent with POPA representing a “housekeeping” enzyme, albeit of unknown function, and POPB being a POP dedicated to cyclic peptide biosynthesis. Despite the greater taxonomic distance between *Galerina* (family Strophariaceae) and *Amanita* (family Amanitaceae) than between *Ab* and *Ap*, POPA of *Gm* more closely aligned with the POPAs of the two *Amanita* species than to its own POPB (Fig. 3). The POPBs showed the same relationship. The clustering of POPBs with each other and POPAs with each other was also true when just the catalytic domains or just the propeller domains were aligned (Additional file 1: Figure S6). This suggests that both domains contribute to the unique catalytic properties of POPB.

### Gene clustering of MSDIN genes and POPB genes

In *Gm*, POPB is clustered with one of the two copies of *AMA1*, encoding the precursor peptide of α-amanitin [8]. A 15-kb genomic lambda clone from the specimen of *Ab* sequenced earlier [1] contained the *AbPOPB* gene and one of the MSDIN family members (variable region GAYPPVPMP, which was present in the earlier *Ab* genome survey sequence but not in the current *Ab* genome) [10]. Otherwise, the current results did not reveal any further evidence of clustering of POPB with any member of the MSDIN family in either *Ab* or *Ap*.

## Discussion

Based on their complete genomes, *Amanita phalloides* (*Ap*) and *A. bisporigera* (*Ab*) together have the genetic capacity to encode more than 50 unique small, cyclic peptides on the same biosynthetic scaffold that they use to biosynthesize the amatoxins and phallotoxins. Additional members of the MSDIN gene family have been described in other species of *Amanita* sect. *Phalloideae*, including *A. ocreata*, *A. exitialis*, *A. rimosa*, and others [1, 6, 18], indicating that collectively this taxon has a large capacity to make a diversity of small peptides. Most of them are predicted to be cyclic but otherwise unmodified, like the cycloamanides, but it is possible that some of them are further modified by, e.g., hydroxylations, like the amatoxins and phallotoxins.

There is little overlap in the complement of MSDIN genes between species. *Ab* and *Ap* have only three MSDIN genes in common (α-amanitin, phallacidin, and Apha_msdin_9/Abis_msdin_20). *Ab* and *A. exitialis* (whose complete genome has not yet been reported) have three known genes in common (α-amanitin, phallacidin, and Abis_msdin_8). *Ap* and *A. exitialis* have four genes in common: α-amanitin, β-amanitin, phallacidin, and Apha_msdin_28. Because the genome assemblies that we have prepared for *Ap* and *Ab* are draft quality assemblies, it is possible that additional MSDIN genes are present in the genomes of the two species.

Surprisingly, the majority of the MSDIN genes found earlier in *Ab* were not present in the current *Ab* genome. The ones in common were α-amanitin, phallacidin, and three unknowns. The most plausible explanation for this is that the two specimens, both identified on morphological criteria as “*A. bisporigera*”, are, in fact, different species. This is supported by comparison of their ITS sequences. Considering the high degree of variation between the MSDIN complement of *Ab* and *Ap* and between both specimens of *Ab* and *A. exitialis*, the degree of variation we observed between the two specimens of *Ab* seems consistent with them being distinct species. This result also suggests that within *Amanita* sect. *Phalloideae* the MSDIN gene family is even larger than our current taxonomic understanding would indicate.

In contrast to *Ab* sensu lato, analyses of the toxins and MSDIN genes of *Ap* are consistent with it being a single, discrete species. Li et al. [6] identified six MSDIN genes by PCR from a specimen of *Ap* collected in Italy. All six genes were present in our isolate of *Ap* collected in California. The genomic similarity between Italian and California isolates of *Ap* is consistent with their similar chemical profiles and the recent introduction of *Ap* from Europe into North America [23, 28].

Of the known chemically characterized cyclic peptides from *Ap*, genes encoding α-amanitin, β-amanitin, phalloidin, phallacidin, and cycloamanide B were present in the *Ap* genome. These five compounds are historically known to be made by specimens of *Ap* from Germany [15, 20]. However, we did not find genes for cycloamanide A, C, and D, nor a gene for the cyclic decapeptide antamanide. The absence of these genes could be due to gaps in our genome assemblies or to natural variation.

Among all the species of *Amanita* whose MSDIN genes have been studied, most members of this family are unique to one species, and only a few are common to more than one. The most widely distributed gene throughout sect. *Phalloideae* encodes α-amanitin. α-Amanitin is highly toxic to insects, mammals, nematodes, and other organisms, and is responsible for >95% of the human deaths from mushroom poisoning [29]. Although the biological rationale for its production is not known, its strong activity and widespread occurrence suggests that it confers a strong selective advantage to the producing fungi. β-Amanitin, phalloidin, and phallacidin are also common, but not universal, in *Amanita* sect. *Phalloideae*. β-Amanitin is as toxic to mammals as α-amanitin, and although the phallotoxins are not toxic to mammals when consumed orally, they might be toxic to other mycovores such as insects.

The genomic information predicted the existence of multiple novel cyclic peptides. Two of them were found in the lipophilic fractions from extracts of *Ap*. The novel compounds, cyclo(SFFFPVP) and cyclo(IVGILGLP), are rich in hydrophobic amino acids. Following the earlier naming of cycloamanides A-D, we have given them the trivial names cycloamanide E and F, respectively. This result is another example demonstrating the utility of genomics for the discovery of novel secondary metabolites [30]. We predict that many of the other MSDIN genes in *Ap*, *Ab*, and other *Amanita* species will also be expressed at the chemical level. Although cycloamanides A, B, and E are not post-translationally modified other than by cyclization, cycloamanides C and D contain oxidized methionine [15]. It is therefore possible that some of the other chemical products of the MSDIN family might also be post-translationally modified, which would make their detection by LC/MS more difficult.

Within the genus *Amanita*, the MSDIN family is found only in section *Phalloideae* [1, 16]. However, α-amanitin is also produced by other agarics including some species of *Galerina* and *Lepiota* [7, 23]. Like *Ab* and *Ap*, *Gm* produces α-amanitin on ribosomes as a 35-amino acid precursor peptide. However, outside the core region itself the primary sequences of the α-amanitin genes of *Gm* and *Ab* are highly divergent, although both are predicted to form amphipathic α-helices in their C-termini (i.e., follower) regions [9]. This conserved secondary structure might be important for correct processing by POPB. Another difference betwen *Gm* and toxin-producing species of *Amanita* is that the genome of *Gm* has only two copies of the gene for α-amanitin and no extended MSDIN-like family [7, 8]. The genes for α-amanitin have not yet been described from any species of *Lepiota*.

Known biological activities of the MSDIN family to date include inhibition of RNA polymerase II (amatoxins), stabilization of F-actin (phallotoxins), immunosuppression (cycloamanides), protection of hepatocytes against phallotoxins and amatoxins (antamanide), and blocking the mitochondrial permeability transition pore (antamanide) [15, 21, 22]. It is unknown what, if any, biological activities the other predicted cyclic peptides in the genomes of *Ap* and *Ab* might have, but their evolutionary persistence suggests that they confer some selective advantage to the producing fungi. Furthermore, the observation that the core regions of the MSDIN family show strong amino acid bias suggests that the core sequences are not mutating randomly. That is, if they were mainly nonexpressed and conferred no advantage to the producing fungi, the core regions would not show any amino acid bias. An adaptive function implies that they have biological activities at the molecular level, which are as yet unknown. The natural function of none of the *Amanita* cyclic peptides are known, but could perhaps protect the fruiting bodies against mycophagy by insects, nematodes, or gastropods.

Currently, only small quantities of the known minor peptides such as the cycloamanides are available because cyclic peptide-producing fungi are obligately ectomycorrhizal and are difficult or impossible to culture [7, 31]. However, it may be possible to make compounds such as cycloamanide E in vitro from 25mer or 35mer linear precursors using the macrocyclase activity of POPB [9].

## Conclusions

Two toxic species of *Amanita *have large but essentially non-overlapping potential for cyclic peptide biosynthesis. The MSDIN family of ribosomally encoded peptides is evolving rapidly in *Amanita *section *Phalloideae*.

## Methods

### Biological materials and nucleic acid extraction

An individual basidiocarp of *A. phalloides* (*Ap*) was collected in Alameda County, California, in the winter of 2011, and an individual basidiocarp of *A. bisporigera* (*Ab*) was collected in Ingham County, Michigan, in the summer of 2010. The *A. bisporigera* specimen used in this work was collected in the same location as the *A. bisporigera* specimen earlier analyzed by pyrosequencing [1]. DNA and RNA were extracted using cetyltrimethyl ammonium bromide (CTAB), phenol, and chloroform and sequenced by Illumina MiSeq technology. RNA from the same specimen of *Ab* was reverse-transcribed and sequenced by Illumina HiSeq technology.

### Sequencing and assembly

Paired-end DNA libraries for both *Ap* and *Ab* were preprocessed to remove sequencing adaptors and low quality reads using Trimmomatic version 0.32 [32]. Leading and trailing low quality bases (below quality score 20) were trimmed, and reads with a length of less than 100 bp were removed. In addition, the three mate-pair libraries (2 kb, 4 kb and 6 kb nominal insert sizes) for *Ap* were trimmed by Trimmomatic and by NextClip version 1.3 [32, 33], removing duplicates and discarding those reads that did not contain the adaptor in either read within the pair. For both *Ap* and *Ab*, reads were assembled using Velvet version 1.2.10 [34] (additional compiling to allow four libraries for *Ap*) with scaffolding enabled for both. The following parameters were used: a kmer length of 99, an expected coverage of 39X, and a coverage cutoff of 9. In addition, for the *Ap* assembly, Gapcloser was also used in gap close mode (asm_flags = 4) [35]. The resulting assemblies were assessed for contamination using the Blobology pipeline (version 20151102) [11]. Raw reads linked to contamination were removed and the remaining reads reassembled with the same parameters. All contigs less than 1 kb were removed. The final assemblies were assessed for completeness using BUSCO version 2 with a beta version of the basidomycete-specfic database in genome mode [12].

For transcript sequences from *Ab*, a paired end RNA-seq library was preprocessed to remove sequencing adaptors and low quality reads using Trimmomatic, requiring a minimum length of 50 bp and trimming leading and trailing low quality bases that had quality scores less than 20. The reads were assembled using Trinity version 2.0.6 with default settings [36].

### Annotation

The genome assemblies of *Ab* and *Ap* were annotated using the MAKER pipeline [12]. A custom repeat library for *Ap* was created and used for repeat masking for both fungi [14]. Publicly available proteins from NCBI for *Amanita muscaria*, *Agaricus bisporus*, and *Laccaria bicolor* along with all manually curated fungal protein sequences from SwissProt were used as evidence to aid gene prediction within MAKER. Trinity transcript assemblies from *Ab* were also used in the annotation of the *Ab* genome. Genes were predicted by Augustus [37], SNAP [38] and GeneMark [39] within the MAKER pipeline, and where multiple predictions were made for a single locus, MAKER picked the prediction that was most concordant with the alignment evidence as the final gene model for that locus. GeneMark was run in both its ES and ET fungal-specific modes. High-quality gene models with transcript or protein alignment support or with predicted proteins containing a Pfam domain were retained for the final annotated gene set.

### Functional annotation

The protein and transcript sequences from the final high-quality gene models were assigned functional annotation using the Trinotate version 2.0.2 pipeline [36]. Functional annotation involved BLAST searches of both the transcripts and proteins against the Swissprot database [40]. Protein domains, signal peptides and transmembane regions were predicted using HMMER v2.3.2 [41], SignalP version 4.1 [42], and tmHMM version 2.0 [43]. The results of these searches were loaded into a Trinotate pre-generated SQLite database, and an annotation report was produced by the report function of Trinotate.

### Annotation of MSDIN and POP genes

Alignments of known MSDIN genes were made within MAKER and used to manually annotate gene models for known and novel MSDIN and MSDIN-like genes. Manual annotation was facilitated with JBrowse [44] for both *Ap* and *Ab* with tracks for MAKER-predicted gene models, protein alignments for *Ab* and *Ap*, and transcript alignments for *Ab* as well as the known MSDIN blastp alignments. For both *Ap* and *Ab* predicted protein sequences from previous studies were used to help guide the gene predictions. When searching using known MSDIN peptide sequences in tblastn, the e-value cutoff was set to 100.

Previously elucidated POPA and POPB protein sequences were aligned within the MAKER pipeline to aid with manual annotation of POP gene models. MAKER gene predictions for POP loci were used as initial gene models that were hand-annotated based on transcript and POP protein alignments. The original MAKER-predicted gene models that overlapped the predicted POP genes were removed, and new manually created gene models were added.

Protein alignments and tree construction were performed using MEGA version 6 [45]. Alignments were performed with ClustalW [46], and the tree was made using the UPGMA method with 500 bootstrap replicates.

All known MSDIN genes whose structures have been confirmed by sequencing of corresponding cDNA molecules have an intron interrupting the fourth from the last codon (including the stop codon), and most have Leu-Cys as the last two amino acids. For *Ab*, transcript evidence was also used to help elucidate intron/exon boundaries within MSDIN genes.

The majority of the *Ab* MSDIN genes had canonical intron acceptor/donor sequences, but the introns of four genes had non-canonical GC-AG acceptor/donor sequences (Abis_msdin_3, 4, 11, and 28). Lengths of the single exon-interrupting intron ranged between 52–58 bp.

### Ortholog and syntolog analysis between *Ap, Ab* and *A. muscaria*

An ortholog search was executed using OrthoMCL version 1.4 [47] comparing the predicted proteins of *Ab* and *Ap* with the published proteins from *Amanita muscaria* [16]. During the alignment step, a BLAST e-value cutoff of 1e-10 was used. The resulting orthoMCL output file was parsed to identify ortholog groups containing genes from all three species, from only two *Amanita* species, or from only a single species.

### Toxin extraction and analysis

Lyophilized *Ap* basidiocarps were frozen in liquid nitrogen, ground with a mortar and pestle to a fine powder, and then extracted by one of two methods. In the first method, the powder was suspended in 50% H_2_O + 40% HPLC-grade methanol + 10% 0.1 M HCl at a concentration of 1 g mushroom tissue/50 ml. Extracts were analyzed by HPLC/MS using an Agilent 1200 HPLC system and a Higgins PROTO 300 C18 5 μm column (250 × 4.6 mm). Mobile phase A was 90% 0.02 M ammonium acetate, pH 5 + 10% acetonitrile, and mobile phase B was 76% 0.02 M ammonium acetate, pH 5 + 24% acetonitrile. The gradient program consisted of 0 to 8% B from 0–4 min, 8 to 18% B from 4–10 min, and 18 to 100% B from 10–30 min. Eluant was monitored by UV absorbance and an in-line Agilent 6120 mass spectrometer in positive ESI mode. Capillary voltage was 5 kV and the drying gas (N_2_) temperature was 350 °C at a flow rate of 12 L/min. The scan range was m/z 580–2000.

In the second method, the powder was resuspended in 90% ethanol at a concentration of 1 g/50 ml. After stirring for one hour at room temperature, the ethanol was removed under vacuum and the residue dissolved in CHCl_3_. The CHCl_3_ was removed by evaporation and the residual oil redissolved in 50% acetontrile. This extract was analyzed using a Waters Xevo G2-XS QToF HPLC/MS/MS interfaced to a Waters Acquity UPLC system. Five microliters were injected onto a BEH C18 UPLC column (2.1 mm × 50 mm, 1.7-μm particle size; Waters Corp.). Column temperature was maintained at 30 °C. The flow rate was 0.3 mL/min with starting conditions at 95% solvent A (10 mM ammonium formate in water) and 5% solvent B (acetonitrile). The 30-min gradient profile for elution was as follows: starting at 5% solvent B and holding for 3 min, then a linear gradient to 99% B at 27 min, holding at 99% B for 1 min to 28 min, at 28.01 min returning to 95% A/5% B, and maintaining until 30 min. The MS settings were electrospray ionization in positive-ion mode, 3 kV capillary voltage, 100 °C source temperature, 350 °C desolvation temperature, 600 L/h desolvation nitrogen gas flow rate, and 35 V cone voltage. Data were acquired using an MS^e^ method having two separate acquisition functions where function 1 was performed with no collision energy and function 2 was performed with a collision energy ramp from 60–100 V. For both functions, the scan range was 50–1500 m/z with a scan rate of 0.2 s per function. Data were analyzed using Masslynx v4.1 (Waters) and mMass v5.5.0 [24].

## Additional file

Additional file 1: Table S1. Maker annotation statistics for *A. phalloides* and *A. bisporigera*. **Table S2** OrthoMCL data comparing *A. phalloides*, A. bisporigera, and *A. muscaria*, showing only the ortholog groups that contain MSDIN or POP genes. Species are shown in parentheses: A.bis = *A. bisporigera*, A.pha = *A. phalloides* and A.mus = *A. muscaria*. **Table S3** Distribution of amino acids in the core regions of the MSDIN peptides in Ab and Ap. Figure 2 shows the results for Ap graphically. **Figure S1** Distribution of MAKER standard gene model GC content for A. phalloides and A. bisporigera. Both species show unimodal GC distribution with peaks at 49 and 48%, respectively. **Figure S2** MS/MS spectrum for the compound eluting at 13.62 min (cycloamanide E). Insert: mMass report for the b-ion series [24]. **Figure S3** MS/MS spectrum for the compound eluting at 14.97 min (cycloamanide F) and mMass report for the b-ion series [24]. **Figure S4** Sequence of the ITS region of *A. phalloides*. **Figure S5** Sequence of the ITS region of *A. bisporigera*. **Figure S6** Phylogenetic tree of the catalytic (cat) and propeller (prop) regions of prolyl oligopeptidases (POPs) from species of *Amanita* and *Galerina*. POPAs are shown in black and POPBs in red. Ab, *A. bisporigera*; Ap, *A. phalloides*; Ath, *A. thiersii*; Am, *A. muscaria*; Gm, *G. marginata*. (DOCX 581 kb)

## Abbreviations

*Ab*: *Amanita bisporigera*
*Ap*: *A. phalloides*
*Gm*: *Galerina marginata*
LC: Liquid chromatography
MS: Mass spectrometry
POP: Prolyl oligopeptidase
ToF: Time of flight
