# Supplementary material for: Expansion and diversification of the MSDIN family of cyclic peptide genes in the poisonous agarics Amanita phalloides and A. bisporigera
Source: BMC Genomics. 2016 Dec 15;17:1038. doi: 10.1186/s12864-016-3378-7 (PMC5159998; doi:10.1186/s12864-016-3378-7)

**Additional file 1 – Pulman et al.**

**Table S1.** Maker annotation statistics for *A. phalloides* and *A.* *bisporigera.*

| Species | Supporting evidence but no Pfam domain^a^ | Pfam domain and supporting evidence | Pfam domain but no supporting evidence | *Ab initio* |
| --- | --- | --- | --- | --- |
| *A. phalloides* | 3381 | 4616 | 180 | 2044 |
| *A. bisporigera* | 9096 | 5377 | 413 | 7303 |

^a^Supporting evidence is transcript or protein alignments for *A. bisporigera* and protein alignments for *A. phalloides*.

**Table S2.** OrthoMCL data comparing *A. phalloides*, *A.* *bisporigera*, and *A.muscaria*, showing only the ortholog groups that contain MSDIN or POP genes. Species are shown in parentheses: A.bis = *A. bisporigera*, A.pha= *A. phalloides* and A.mus = *A.muscaria.*

**Table S3.** Distribution of amino acids in the core regions of the MSDIN peptides in *Ab* and *Ap*. Fig. 2 shows the results for *Ap* graphically.

|  | **total number in *Ap*** | **% of total in *Ap*** | **total number in *Ab*** | **% of total in *Ab*** |
| --- | --- | --- | --- | --- |
| **Ser** | 7 | 2.2 | 9 | 3.1 |
| **Phe** | 41 | 13.0 | 27 | 9.3 |
| **Thr** | 6 | 1.9 | 1 | 0.4 |
| **Asn** | 3 | 1.0 | 3 | 1.0 |
| **Lys** | 1 | 0.3 | 0 | 0 |
| **Glu** | 1 | 0.3 | 7 | 2.4 |
| **Tyr** | 8 | 2.5 | 10 | 3.5 |
| **Val** | 8 | 2.5 | 7 | 2.4 |
| **Gln** | 3 | 1.0 | 3 | 1.0 |
| **Met** | 3 | 1.0 | 9 | 3.1 |
| **Cys** | 8 | 2.5 | 6 | 2.1 |
| **Leu** | 35 | 11.1 | 27 | 9.3 |
| **Ala** | 18 | 5.7 | 10 | 3.5 |
| **Trp** | 12 | 3.8 | 13 | 4.5 |
| **Pro** | 98 | 31.1 | 88 | 30.5 |
| **His** | 1 | 0.3 | 0 | 0 |
| **Asp** | 4 | 1.3 | 6 | 2.1 |
| **Ile** | 38 | 12.1 | 42 | 14.5 |
| **Arg** | 8 | 2.5 | 7 | 2.4 |
| **Gly** | 12 | 3.8 | 14 | 4.8 |
| **Total** | 315 | 100 | 289 | 100 |

**Fig. S1.** Distribution of MAKER standard gene model GC content for *A. phalloides* and *A.* *bisporigera*. Both species show unimodal GC distribution with peaks at 49% and 48%, respectively.

**
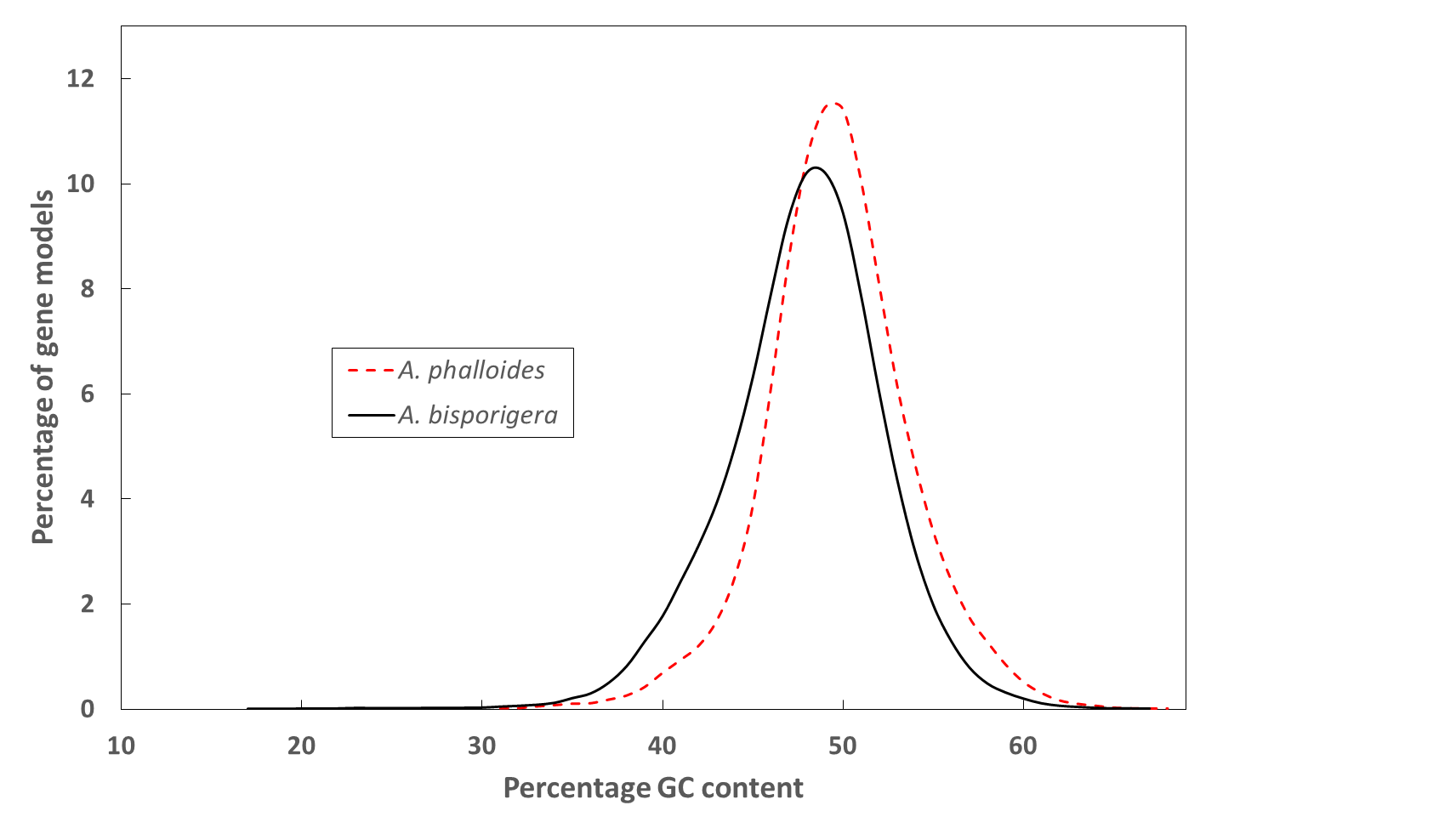
**

**Fig. S2.** MS/MS spectrum for the compound eluting at 13.62 min (cycloamanide E). Insert: mMass report for the b-ion series [24].


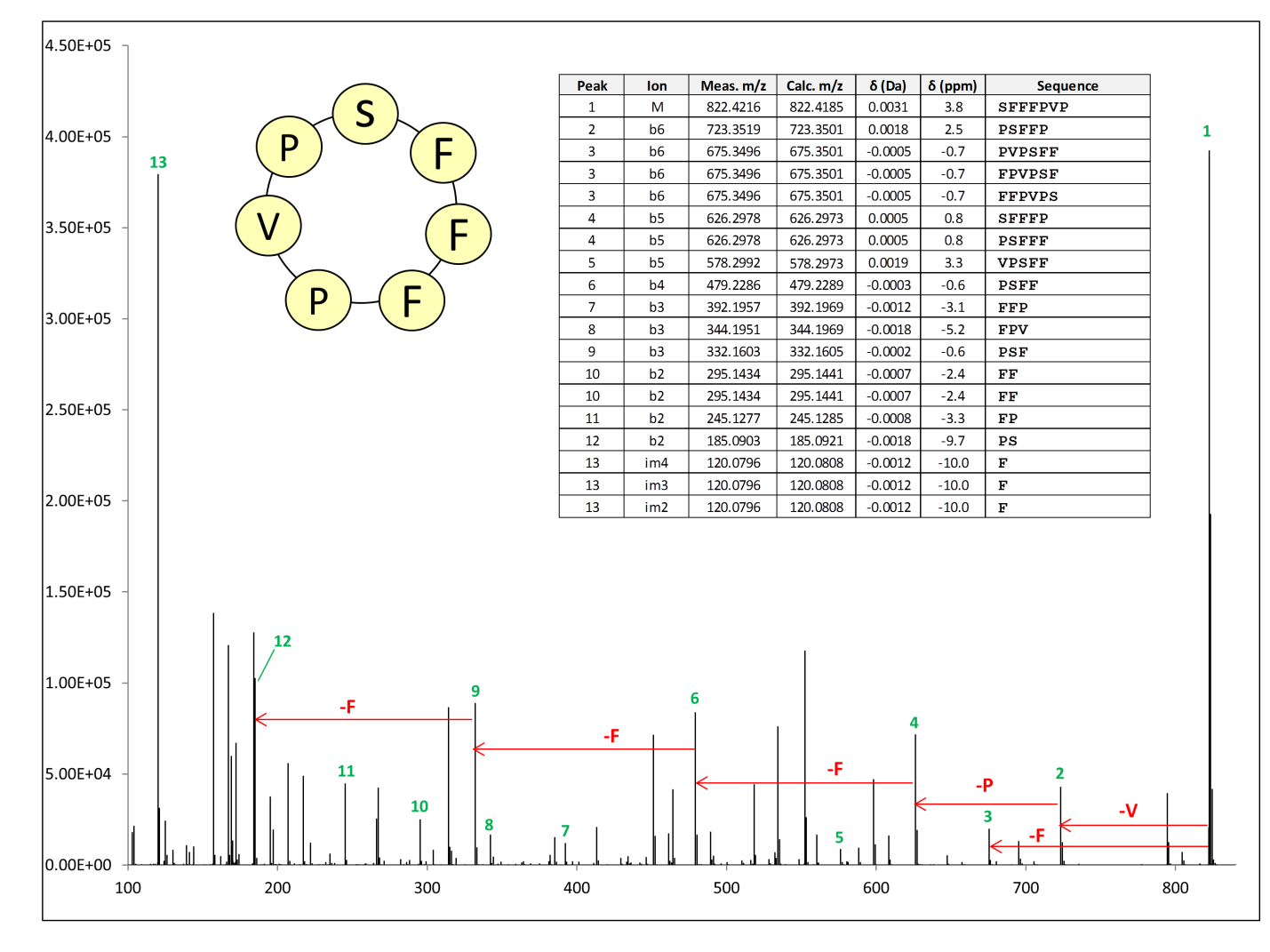


**Fig. S3.** MS/MS spectrum for the compound eluting at 14.97 min (cycloamanide F) and mMass report for the b-ion series [24].


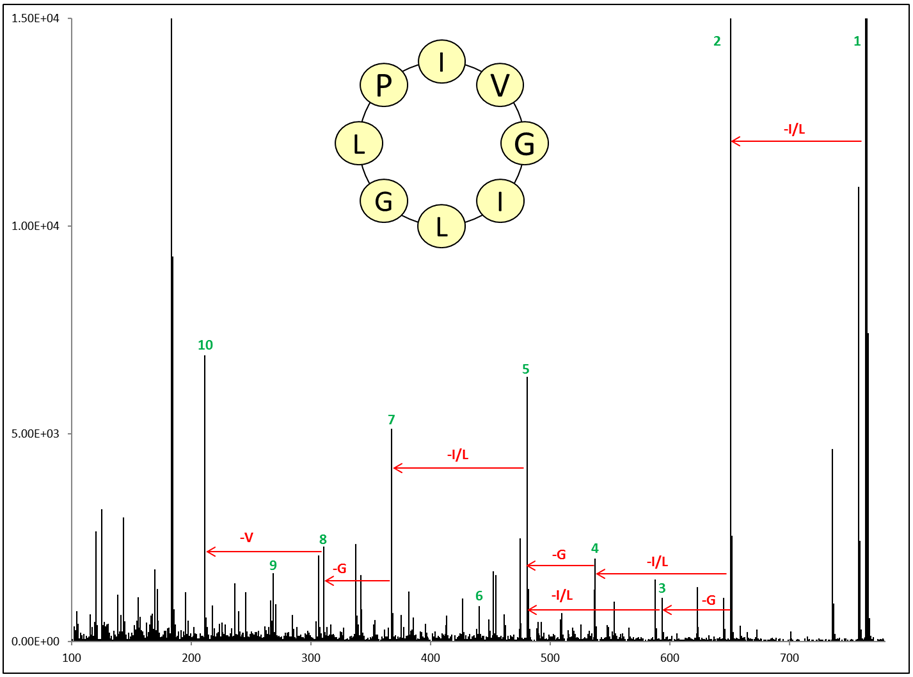

**Fig. S4.** Sequence of the ITS region of *A. phalloides.*

tggtcattaccaattccacctgtgcacacttgtagacacttgggaatgagagaccttgac

cagtctcttgagaagttgaaaatctgggtgtctatgccattttattaaacactagttgca

tgtttatagaatgatgatttgattaaatataaagtacaactttcaacaacggatctcttg

gctctcgcatcgatgaagaacgcagcgaaatgcgataagtaatgtgaattgcagaattca

gtgaatcatcgaatctttgaacgcaccttgcgctccttggcattccgaggagcatgcctg

tttgagtgtcattaaattctcaagacctgtctgcttttttgataggtattggatttttgg

gggttgcaggctgtttcaaataaaaatagccttgctctctttgaatgtattagtggagaa

aagccattgaactccattggtgtgataaaacctatcaatgccaggagcaatatcacttct

ctctgctgtctaactgtgactgtctgtataaatttatatggatggggacaacttgaccaa

cttgacctcaaatcaggtaggactacccgctgaacttaagcatatcaataagcggaggaa

**Fig. S5.** Sequence of the ITS region of *A. bisporigera.*

GATCATTAATGAGATGAACCTTGAGGCTGTAGCTGGCCCATCTGGGCATGTGCACGTCTCTGGTCATTAC

CAATTCCACCTGTGCACACTTGTAGACACTTGGGAATGAGAGACTTTGACCGGTCTCTTGAGAAGTTGAA

ATCTAGGTGTCTATGCCATTTTATTAAACACTAGTTGCATGTTTATAGAATGATGATTTGATTATATATA

AAGTACAACTTTCAACAACGGATCTCTTGGCTCTCGCATCGATGAAGAACGCAGCGAAATGCGATAAGTA

ATGTGAATTGCAGAATTCAGTGAATCATCGAATCTTTGAACGCACCTTGCGCTCCTTGGCATTCCAAGGA

GCATGCCTGTTTGAGTGTCATTAAAGTCTCAAGACCTGTCTGATTTTGATAGGTATTGGATTTTGGGGGT

TGCAGGCTTTTTCAGATAGCCTGCTCTCCTTGAATGTATTAGTGGAGAAAGAGCCATTTGAACTCCATTG

GTGTGATAAAACCTATCAATGCCAGGAGCAATGTTAGTTTTCTCTGCTGTCTAACTGTCTGTAAAAATGG

ACAATTTGACCAACTTGACCTCAAATCAGGTAGGACTACCCGCTGAACTTAAGCATATC

**Fig. S6**. Phylogenetic tree of the catalytic (cat) and propeller (prop) regions of prolyl oligopeptidases (POPs) from species of *Amanita* and *Galerina*. POPAs are shown in black and POPBs in red. Ab, *A. bisporigera*; Ap, *A. phalloides*; Ath, *A. thiersii*; Am, *A. muscaria*; Gm, *G. marginata*.


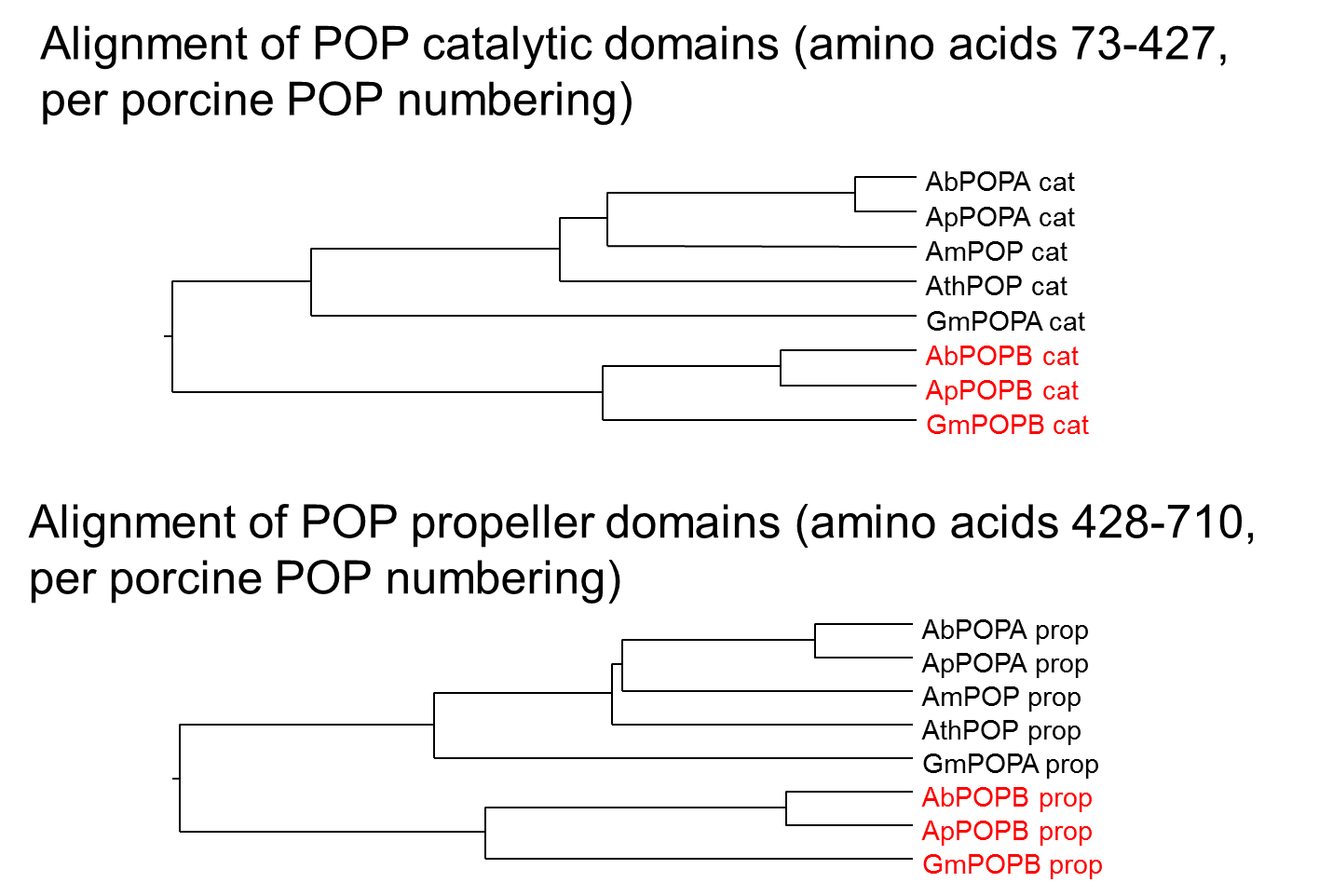

Supplement: Additional file 1: Table S1. — Maker annotation statistics for A. phalloides and A. bisporigera. Table S2 OrthoMCL data comparing A. phalloides, A. bisporigera, and A. muscaria, showing only the ortholog groups that contain MSDIN or POP genes. Species are shown in parentheses: A.bis = A. bisporigera, A.pha = A. phalloides and A.mus = A. muscaria. Table S3 Distribution of amino acids in the core regions of the MSDIN peptides in Ab and Ap. Figure 2 shows the results for Ap graphically. Figure S1 Distribution of MAKER standard gene model GC content for A. phalloides and A. bisporigera. Both species show unimodal GC distribution with peaks at 49 and 48%, respectively. Figure S2 MS/MS spectrum for the compound eluting at 13.62 min (cycloamanide E). Insert: mMass report for the b-ion series [24]. Figure S3 MS/MS spectrum for the compound eluting at 14.97 min (cycloamanide F) and mMass report for the b-ion series [24]. Figure S4 Sequence of the ITS region of A. phalloides. Figure S5 Sequence of the ITS region of A. bisporigera. Figure S6 Phylogenetic tree of the catalytic (cat) and propeller (prop) regions of prolyl oligopeptidases (POPs) from species of Amanita and Galerina. POPAs are shown in black and POPBs in red. Ab, A. bisporigera; Ap, A. phalloides; Ath, A. thiersii; Am, A. muscaria; Gm, G. marginata. (DOCX 581 kb) [file 12864_2016_3378_MOESM1_ESM.docx]
